# Network Pharmacology to Explore the Molecular Mechanisms of *Prunella vulgaris* for Treating Hashimoto’s Thyroiditis

**DOI:** 10.3389/fphar.2021.700896

**Published:** 2021-10-06

**Authors:** Xiao-xiong Gan, Lin-kun Zhong, Fei Shen, Jian-hua Feng, Ya-yi Li, Si-jing Li, Wen-song Cai, Bo Xu

**Affiliations:** ^1^ Department of Thyroid Surgery, Guangzhou First People’s Hospital, School of Medicine, South China University of Technology, Guangzhou, China; ^2^ Department of General Surgery, Zhongshan City People’s Hospital Affiliated to Sun Yat-sen University, Zhongshan, China

**Keywords:** *Prunella vulgaris*, Hashimoto’s thyroiditis, network pharmacology, molecular docking, anti-inflammatory response

## Abstract

**Purpose:** *Prunella vulgaris* (*PV*), a traditional Chinese medicine, has been used to treat patients with thyroid disease for centuries in China. The purpose of the present study was to investigate its bioactive ingredients and mechanisms against Hashimoto’s thyroiditis (HT) using network pharmacology and molecular docking technology to provide some basis for experimental research.

**Methods:** Ingredients of the *PV* formula were retrieved from the Traditional Chinese Medicine Systems Pharmacology (TCMSP) database. Additionally, HT-related genes were retrieved from the UniProt and GeneCards databases. Cytoscape constructed networks for visualization. A protein–protein interaction (PPI) network analysis was constructed, and a PPI network was built using the Search Tool for the Retrieval of Interacting Genes (STRING) database. These key targets of *PV* were enriched and analyzed by molecular docking verification, Gene Ontology (GO), and Kyoto Encyclopedia of Genes and Genomes (KEGG) enrichment.

**Results:** The compound–target network included 11 compounds and 66 target genes. Key targets contained Jun proto-oncogene (*JUN*)*,* hsp90aa1.1 (*AKI*)*,* mitogen-activated protein kinase 1 (*MAPK1*)*,* and tumor protein p53 (*TP53*). The main pathways included the AGE-RAGE signaling pathway, the TNF signaling pathway, the PI3K–Akt signaling pathway, and the mitogen-activated protein kinase signaling pathway. The molecular docking results revealed that the main compound identified in the *Prunella vulgaris* was luteolin, followed by kaempferol, which had a strong affinity for HT.

**Conclusion:** Molecular docking studies indicated that luteolin and kaempferol were bioactive compounds of *PV* and might play an essential role in treating HT by regulating multiple signaling pathways.

## Introduction

*Prunella vulgaris* (*PV*) is a perennial herbaceous plant in the genus *Prunella*1. It is a Chinese medicine widely used to treat inflammation, eye pain, and headaches (Wang et al., 2019; Chen et al., 2020). The anti-inflammatory effects of *PV* have been recognized during the long-term practice of traditional Chinese medicine (TCM) (hui ZRGwsbydwy, 2000). Currently, *PV* is combined with Western medicines, such as levothyroxine, indomethacin, or prednisone, in liquid or capsules and has been used to treat Hashimoto’s thyroiditis patients. It has been shown that *PV* significantly reduces the antibody titers of thyroid peroxidase antibody (TPO-Ab) and thyroglobulin antibody (TG-Ab) (Yang et al., 2007; Zhang, 2014). However, the potential underlying mechanisms by which *PV* might exert its anti-inflammatory effects are poorly understood.

Hashimoto’s thyroiditis (HT) is a genetic autoimmune disorder characterized by the destruction of thyroid cells by cell- and antibody-mediated immune responses (Hu et al., 2019). In developed countries, HT is the most common cause of hypothyroidism. The estimated incidence of HT is 3.5 per 1,000 per year in women and 0.8 per 1,000 per year in men (Ala et al., 2015). Effective treatment options for HT are limited. The main method and purpose of HT treatment is the control of hypothyroidism and consists of oral administration of a synthetic hormone, levothyroxine 4 (L-T4) (Wiersinga and Wilmar, 2001).

Additionally, the association between vitamin D deficiency, HT pathogenesis, and thyroid hypofunction has been demonstrated in several studies (Liontiris and Mazokopakis, 2017; Roehlen et al., 2018; Chao et al., 2020). Therefore, due to the low cost and minimal side effects of vitamin D, monitoring and supplementation in patients with HT may be recommended (Liontiris and Mazokopakis, 2017). Surgical therapy can be recommended for patients with HT concurrent with nodules or malignancies (Caturegli et al., 2013). However, HT patients commonly have a higher prevalence rate of postoperative complications than thyroid disorders (Gan et al., 2021).

Network pharmacology, combined with pharmacology and pharmacodynamics, is a novel research field that clarifies numerous compounds’ synergistic effects and underlying mechanisms by analyzing various networks of complex and multilevel interactions (Cao et al., 2018). The study explored the potential pharmacodynamic material basis and molecular mechanism of PV against HT using network pharmacology and molecular docking technology and predicted their potential targets and signaling pathways.

## Materials and Methods

### Bioactive Compound Identification Screening

The active constituents of *PV* were obtained from the Traditional Chinese Medicine Systems Pharmacology Database, the Analysis Platform (TCMSP, http://tcmspw.com/) database, and subsequent network pharmacology (Tan et al., 2017). The TCMIP database of Chinese herbal medicines is based on the Chinese Pharmacopoeia (2015 edition), which contains 500 kinds of Chinese herbal medicines and 30,069 ingredients (Ru et al., 2014). The names and aliases of herbs and ingredients were used as keywords. Then, two parameters of ADME (the absorption, distribution, metabolism, and excretion screening method) drug-likeness (DL) and oral bioavailability (OB) were used to predict the bioactive compounds. In drug discovery and development processes, absorption, distribution, metabolism, and excretion (ADME) evaluations are necessary to predict biologically active compounds (Su et al., 2007). In this study, OB > = 30% and DL quality > = 0.18 were selected as criteria for screening the active compounds identified within the TCMSP database (Ning et al., 2017).

### Identification of the Direct Protein Targets

The potential targets for the components of *PV* were retrieved from both TCMSP databases (http://lsp.nwsuaf.edu.cn/tcmsp.php), including 6,511 drug molecules and nearly 4,000 targets as well as the interaction between them (Ru et al., 2014). The UniProt Knowledgebase (UniProtKB) is a protein database partially curated by experts and contains 54, 247, 468 sequence entries (Zhang et al., 2020). Gene information, including the gene name and the gene ID, was confirmed by the UniProt database (https://www.uniprot.org).

### Predicting the Targets of HT

The GeneCards database (https://www.genecards.org/) and selection according to the criterion of RiskScore >1, the Therapeutic Target Database (TTD, https://db.idrblab.org/ttd/), OMIM (https://omim.org/), PharmGkb (https://www.pharmgkb.org/), and DrugBank (https://www.drugbank.ca/) were used to collect information on HT related to target genes. The association of *PV* with HT was then gathered as the core targets of *PV* for HT.

### Construction of the Component–Target Gene Network

In this research, the network of component–target interactions was established, and the interaction between active compounds and their core target proteins was ascertained by Cytoscape 3.7.2 (http://www.cytoscape.org/) (Wang et al., 2011). Moreover, it was visualized by Cytoscape software, an open-source platform for visualizing complex networks (Shannonand et al., 2003). In the network, nodes represent the herbal medicines, active phytochemical compounds, targets, or signaling pathways, while edges represent the interactions between the nodes (Barabási and Oltvai, 2004). The top two compounds were chosen as the ligand for molecular docking. The degree of a node represents the number of connections (edges) that this node has with the rest of the network (Tang et al., 2020). The larger the quantitative value, the more important the node in the network, and the more likely the component is the *PV* of the key ingredient.

### Construction of the Protein–Protein Interaction (PPI) Network

The candidate targets of *PV* for HT treatment were imported into the STRING database (https://string-db.org/) (Consortium UP, 2010) to construct a PPI network. The network analysis plug-in in Cytoscape software was used to analyze network topological features to screen the hub nodes in the PPI network (Saito et al., 2012). Degree centrality denotes several direct connections of a node to all other nodes in the network. The value of degree was used as a reference for the importance of the core target by the CytoNCA, a plug-in of Cytoscape for network centrality analysis.

### GO Functional Enrichment and KEGG Pathway Analysis

Gene Ontology (GO) functional annotation and Kyoto Encyclopedia of Genes and Genomes (KEGG) pathway enrichment were performed in R using the ClusterProfiler package, and p. adjust (FDR) < 0.05 was considered statistically significant (Yu et al., 2012).

### Target Screening by Molecular Docking

The three-dimensional (3D) molecular structures of hsp90aa1.1 (*AKI*) (PDB ID: P31749) (Wu et al., 2010) and mitogen-activated protein kinase 1 (MAPK1) (PDB ID: P28482) (Ward et al., 2017) were downloaded from the RCSB Protein Data Bank (http://www.rcsb.org/pdb). The protein structure was processed by AutoDock Tools (Morris et al., 2009) to remove water molecules and connect hydrogen atoms, and then Gasteiger charges were added to the ligands and the protein templates and saved as a PDBQT file. Two-dimensional (2D) structures of luteolin and kaempferol were downloaded from PubChem (available online: https://pubchem.ncbi.nlm.nih.gov/) as. sdf. Luteolin and kaempferol were vital active compounds, and *AKI* and *MAPK1* were considered the major targets. Molecular docking of the ligands luteolin and kaempferol to the *AKI* and *MAPK1* protein active sites was carried out using AutoDock Vina 1.1.2. Each docking calculation generated 20 structures, and the molecular docking output was prioritized based on the frequency of the possible ligand-binding site and a free energy score. Finally, the best possible conformations and visualized using Discovery Studio Visualizer 2.5 and PyMOL software (PyMol Molecular Graphics System, Version 1).

## Results

### Active Compounds of PV

**FIGURE 1:**
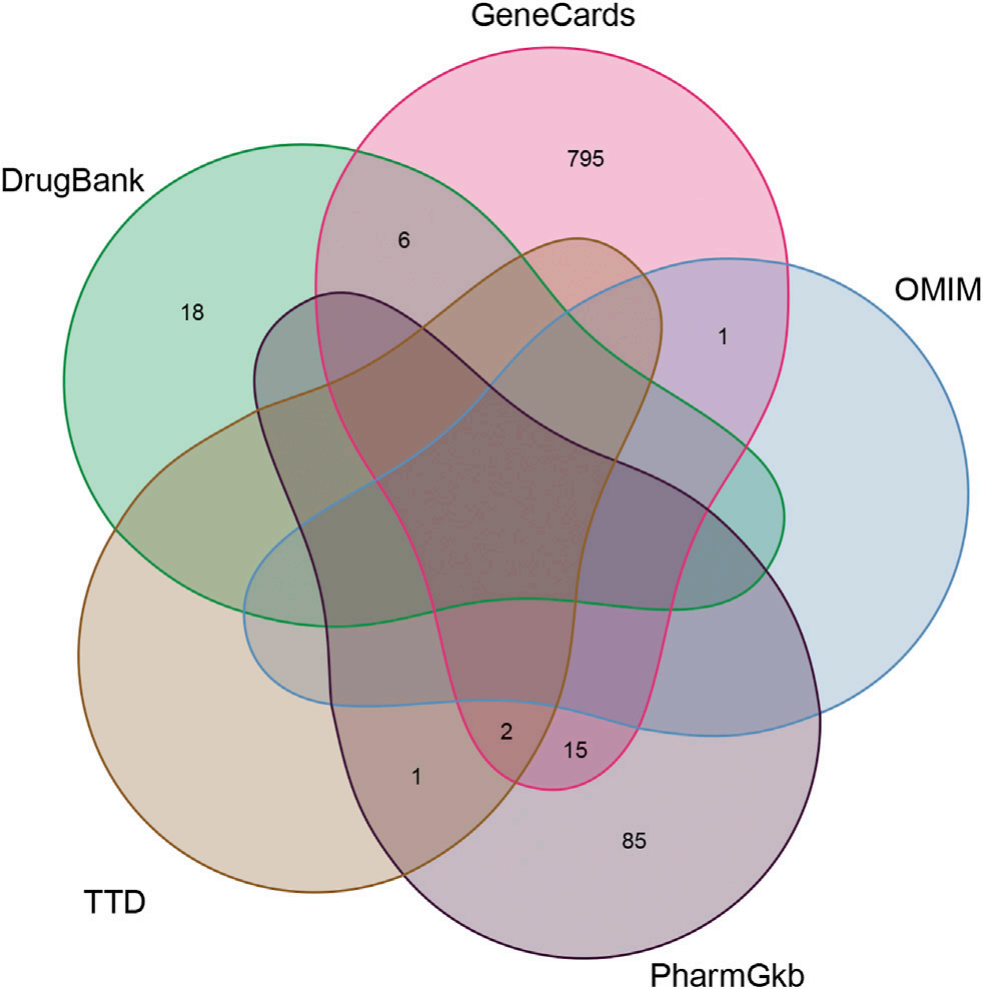
The Venn diagram of *PV* targets.

**FIGURE 2:**
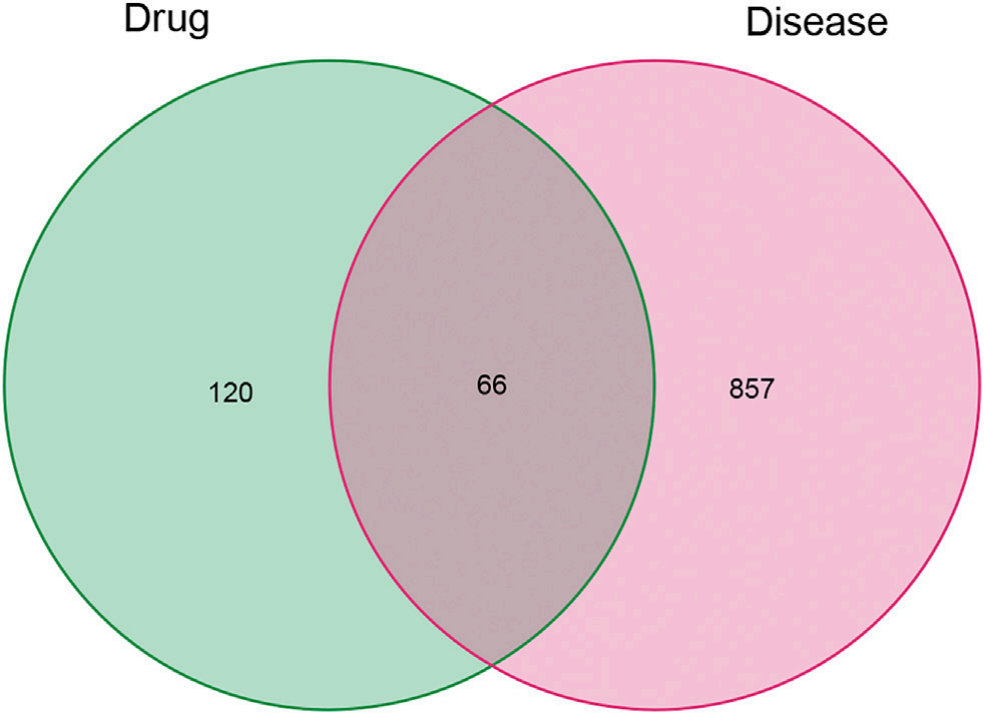
Venn plot showing the intersections of herbs and HT-related genes.

**FIGURE 3:**
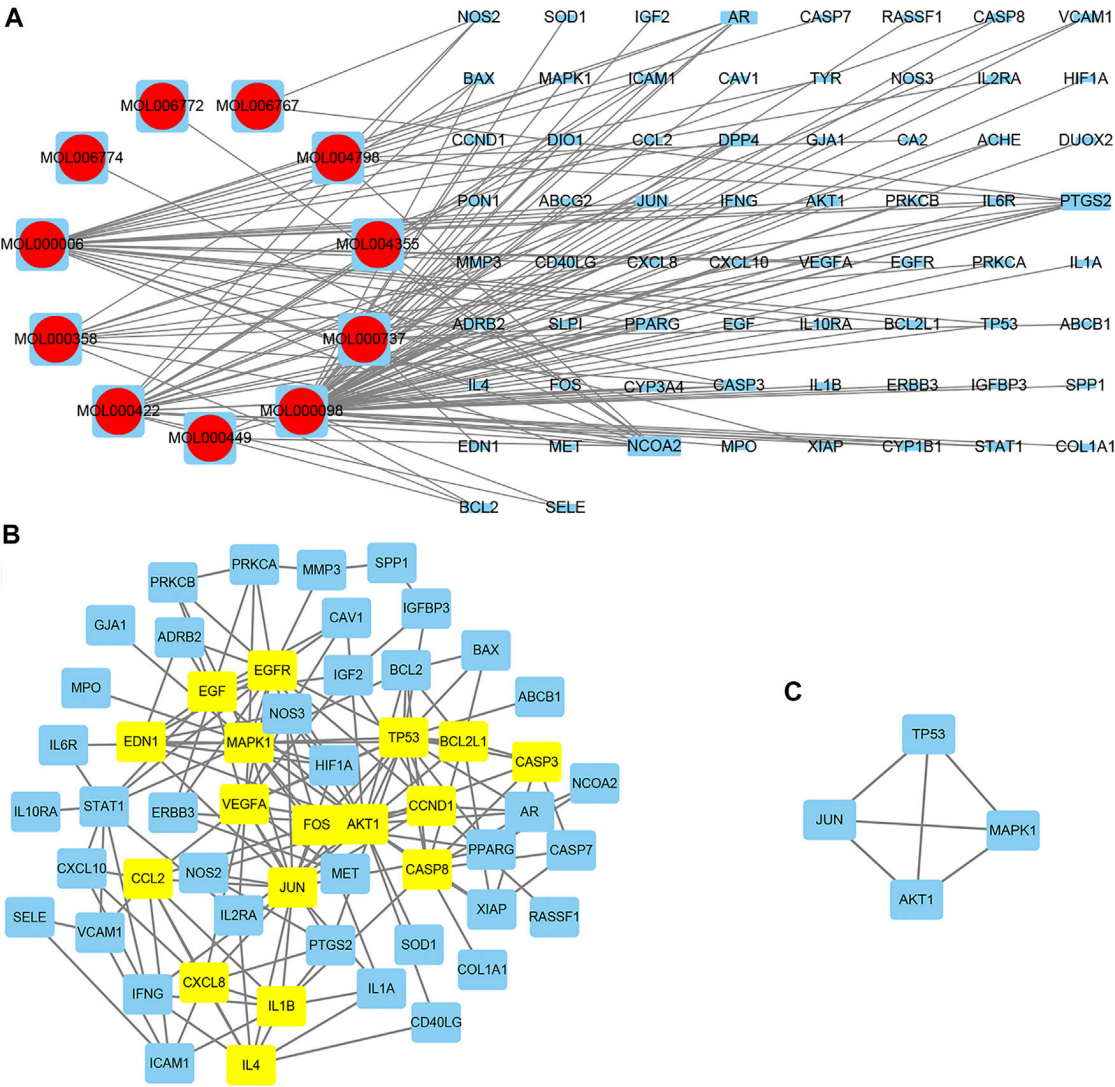
The herb–compound–target network.

Of these, 923 were targets of the herbs comprising *PV*, and the Venn diagram of *PV* targets is shown in Figure 1. A Venn plot showing the intersections of herbs and HT-related genes is shown in Figure 2. The main active components of *PV* containing 11 compounds are shown in Table 1, and the structures are shown in Table 2. Key targets containing Jun proto-oncogene (*JUN*)*, AKI, MAPK1,* and tumor protein p53 (*TP53*) are shown in Table 3 and Figure 3C. Moreover, the scores of four centralities were calculated by CytoNCA, and details are provided in Table 4.

**TABLE 1:** Basic information of some active components of *Prunella vulgaris*.

**Mol ID**	**Chemical component**	**OB (%)**	**DL**
MOL000006	Luteolin	36.16	0.25
MOL000098	Quercetin	46.43	0.28
MOL000358	Beta-sitosterol	36.91	0.75
MOL000422	Kaempferol	41.88	0.24
MOL000449	Stigmasterol	43.83	0.76
MOL000737	Morin	46.23	0.27
MOL004355	Spinasterol	42.98	0.76
MOL004798	Delphinidin	40.63	0.28
MOL006767	Vulgaxanthin-I	56.14	0.26
MOL006772	Poriferasterol monoglucoside_qt	43.83	0.76
MOL006774	Stigmast-7-enol	37.42	0.75

**TABLE 2:** Information for candidate targets from compounds of *Prunella vulgaris*.

**Molecule name**	**Structure**
Luteolin	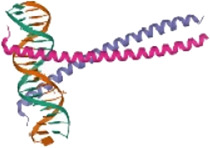
Quercetin	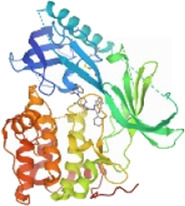
Beta-sitosterol	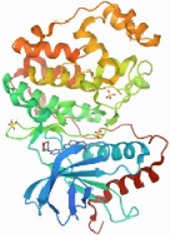
Kaempferol	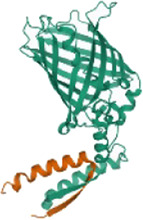
Stigmasterol	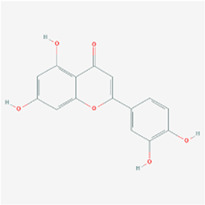
Morin	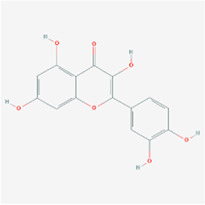
Spinasterol	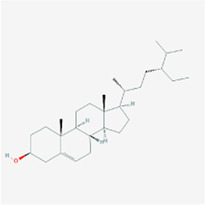
Delphinidin	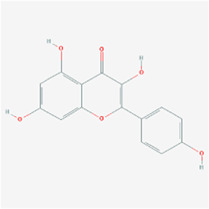
Vulgaxanthin-I	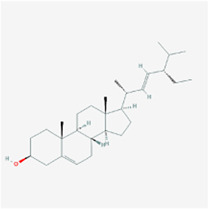
Poriferasterol monoglucoside_qt	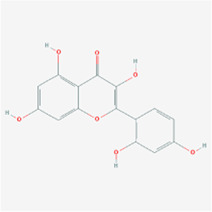
Stigmast-7-enol	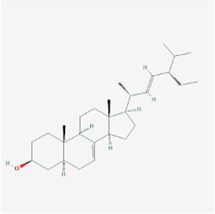

**TABLE 3:** Information for targets gene from compounds of *Prunella vulgaris*.

**Gene name**	**Code**	**Structure**
JUN	P05412	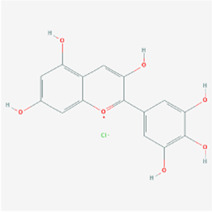
AKI	P31749	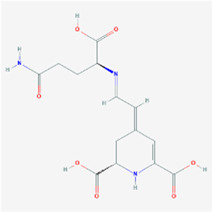
MAPK1	P28482	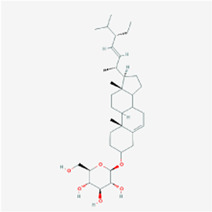
TP53	P04637	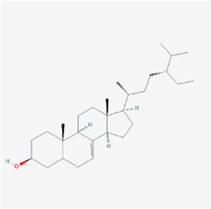

**TABLE 4:** The topological parameters of hub targets.

**Target**	**Degree**	**Betweenness**	**Closeness**
JUN	11	51.61	0.76
TP53	10	28.12	0.73
AKT1	9	24.83	0.70
MAPK1	8	13.84	0.67
EGFR	7	9.07	0.59
VEGFA	7	19.16	0.64
FOS	6	6.04	0.62
CCL2	6	6.44	0.57
CXCL8	5	2.54	0.55
BCL2L1	5	1.25	0.53
CASP8	5	9.35	0.57
IL-1B	5	9.15	0.55
CCND1	5	1.57	0.59
EDN1	5	3.45	0.59
EGF	4	0.83	0.52
IL-4	4	0.00	0.50
CASP3	4	0.75	0.52

### The Construction of Herb–Compound–Target Network

The herb–compound–target network contained 77 nodes (11 compounds and 66 genes), as shown in Figure 3. The node size represents the importance of a node, and the bigger size indicates more importance. According to the degree of the compound, we finally chose the eleven more important compounds, including MOL000006 (luteolin), MOL000098 (quercetin), MOL000358 (beta-sitosterol), MOL000422 (kaempferol), MOL000449 (stigmasterol), MOL000737 (morin), MOL004355 (spinasterol), MOL004798 (delphinidin), MOL006767 (vulgaxanthin-I), MOL006772 (poriferasterol monoglucoside_qt), and MOL006774 (stigmast-7-enol), and the details are shown in Table 1 and Table 2.

### Prediction Results of Disease Targets and the Construction of the PPI Network

**FIGURE 4:**
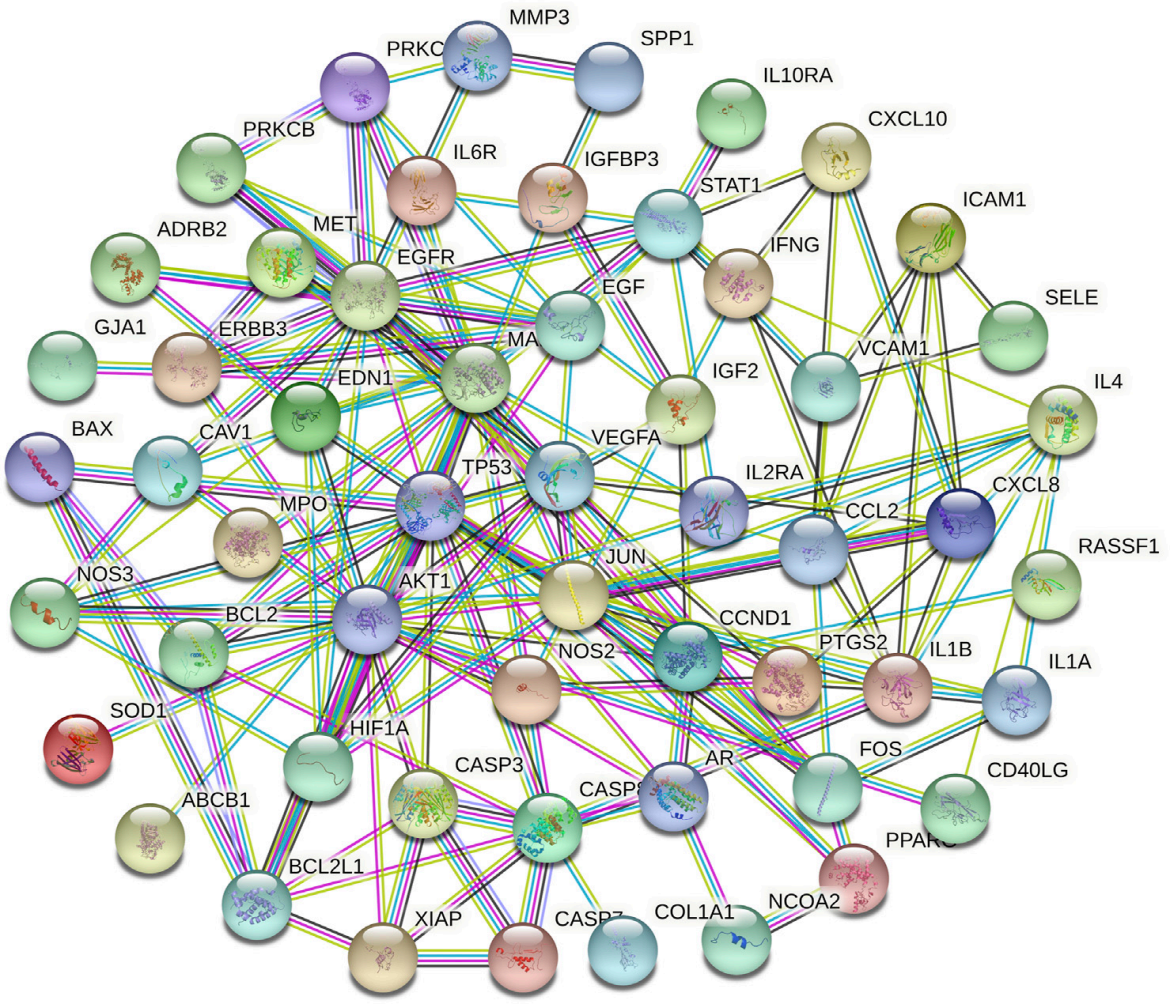
The PPI networks of relative targets.

A total of 66 disease-related targets, 11 *PV*-related targets, and 17 intersection targets were identified (Figures 3A,4). Our study showed that targets had strong relationships in the PPI network. There were 17 nodes in the PeLBD protein interaction network, which were the core targets of *PV* in the treatment of HT (Table 4). *JUN, AKI, MAPK1*, and *TP53* (Aziz et al., 2018) were considered hub genes (Figure 3B).

### GO and KEGG Enrichment Analyses

**FIGURE 5:**
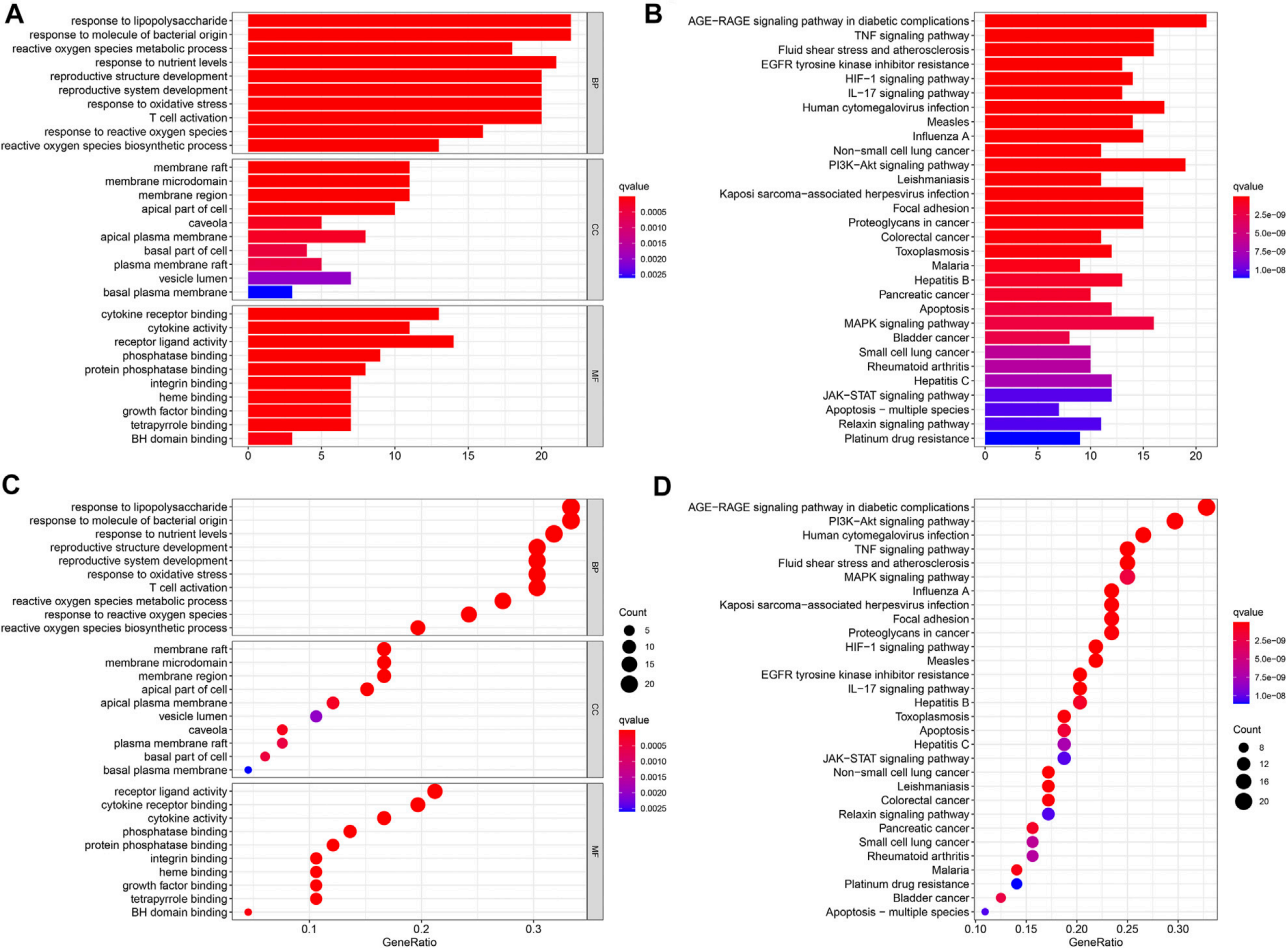
GO and KEGG functional annotation pathway enrichments. The barplot **(A, B)** and the bubble **(C, D)**. The KEGG signaling pathways.

**FIGURE 6:**
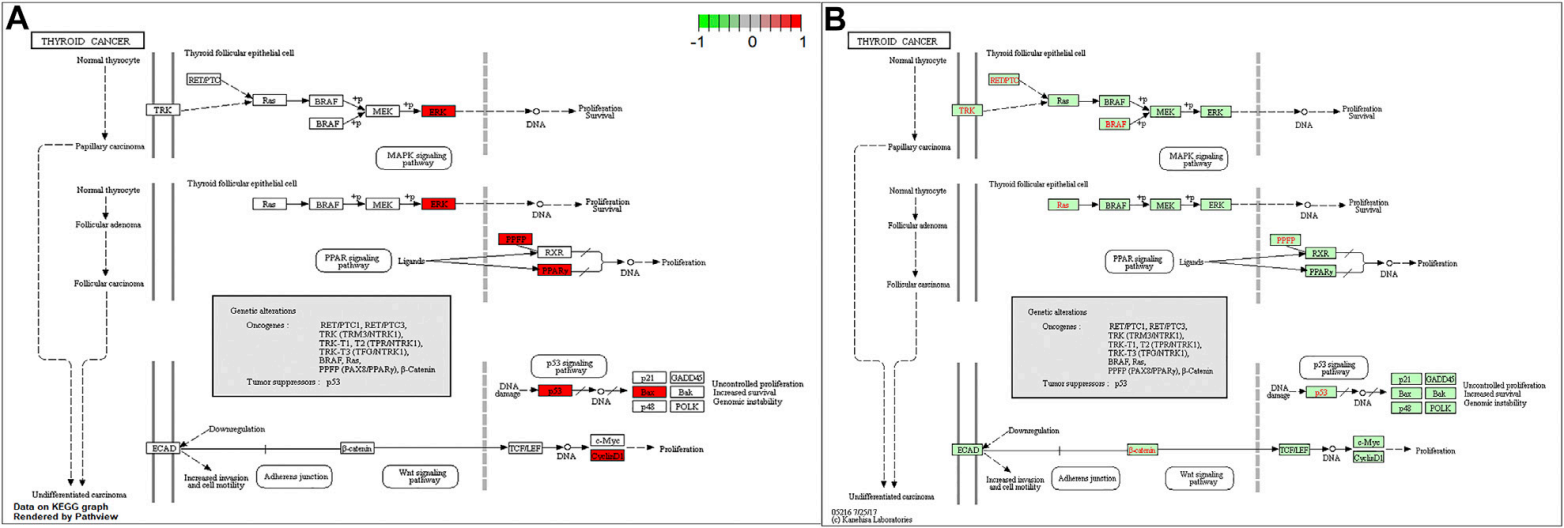
The KEGG signaling pathways.

To better understand the function of intersecting genes, we conducted an enrichment analysis of Gene Ontology (GO). The top 10 biological processes mainly an inflammatory response, a cell-to-cell reaction, and a metabolic process are ranked in Figures 5A, C. In the enrichment analysis of GO pathways, cellular components mainly contained membrane rafts, membrane microdomains, and membrane regions. Meanwhile, the GO terms enriched for a molecular function were mainly cytokine receptor binding, cytokine activity, and receptor-ligand activity. Furthermore, the KEGG pathway enrichment analysis was also conducted. A total of 30 top-ranking pathways (Figures 5B, D) were identified (*p* < 0.05). The relative enrichment analysis showed the following pathways: the AGE-RAGE signaling pathway in diabetic complications, the TNF signaling pathway, the PI3K–Akt signaling pathway, pathways in cancer, the MAPK signaling pathway, and the cancer-associated pathways, as shown in Figures 5B and Figure 6.

### Results of Molecular Docking

**FIGURE 7:**
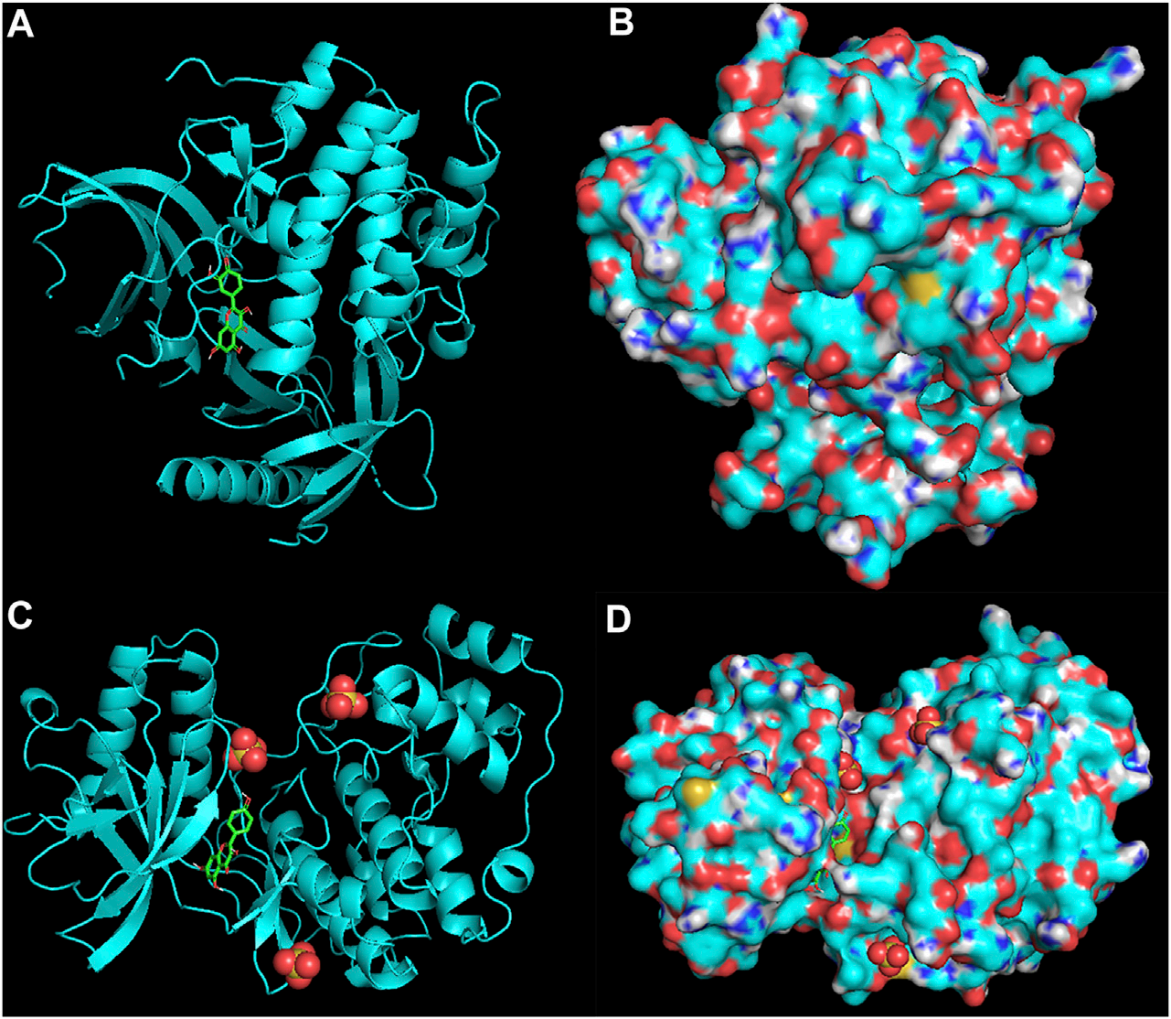
AKI protein: luteolin **(A,B)** and MAPK1 protein: kaempferol **(C,D)**.

The molecular docking assay showed that the *AKI* and *MAPK1* proteins have a stronger affinity for HT disease molecules. Luteolin (MOL000006) was considered as the uppermost active ingredient of PV against HT. Meanwhile, there was also a strong association between kaempferol (MOL000422) and *AKI* with *MAPK1*. The *AKI* and *MAPK1* protein active pockets showed that the small molecules luteolin and kaempferol have a compact binding pattern (Figure 7).

## Discussion

The precise pathogenesis of HT remains unclear. HT is the most prevalent autoimmune thyroid disorder; currently, there is no effective means of preventing and treating HT. Hashimoto’s thyroiditis (HT) is usually manageable by levothyroxine (L-T4) administration, reducing the thyroid volume and supplementing the lack of hormones. Despite thyroid hormone replacement, some euthyroid patients with HT will continue to experience persistent symptoms that reduce their quality of life. *PV* has been empirically used to treat thyroid disorders, including HT in TCMSP, which has been applied for thousands of years. Some of its basic mechanism remains unknown. Recent studies have shown that *PV* plays an essential role in reducing the titers of TPO-Ab, TG-Ab, and Th17 cells in autoimmune and inflammatory disorders, including HT (Wang et al., 2019; hui ZRGwsbydwy, 2000; Yang et al., 2007; Zhang, 2014; Hu et al., 2019; Ala et al., 2015; Wiersinga and Wilmar, 2001). In addition, several recent studies have demonstrated that the anti-inflammatory effect of *PV* is related to NF-κB in stimulated macrophages (Wu et al., 2010; Yu et al., 2012). This study demonstrates that *PV* has a potential therapeutic effect on HT and could explore novel anti-inflammatory therapies for its treatment.

Studies have suggested that the innate immune response in thyrocytes facilitates autosensitization, which may eventually lead to thyroid autoimmunity (Akira et al., 2011; Kawashima et al., 2013). In the study, eleven main active components were screened of *PV*: luteolin, quercetin, beta-sitosterol, kaempferol, stigmasterol, morin, spinasterol, delphinidin, vulgaxanthin-I, poriferasterol monoglucoside_qt, and stigmast-7-enol. Molecular docking showed that the active ingredients, including luteolin and kaempferol, had a good affinity for the hub disease proteins in clinical therapeutics (Xia et al., 2016; Habza-Kowalska et al., 2019). The results demonstrated that luteolin has a strong affinity for disease proteins of HT, and quercetin had a strong affinity for serum thyroid peroxidase (TPO). These results indicated that luteolin and kaempferol might play some important roles in the treatment of HT. Flavonoids are a large group of plant‐derived compounds, and it is well established that certain flavonoids exhibit anti‐inflammatory properties (Lee et al., 2017). Luteolin is one of the most common flavones with antioxidant, anticancer, anti-inflammatory, and antiapoptotic properties in TCMSP (De Stefano et al., 2021). A previous study also suggests that kaempferol is a potential bioaccessible TPO activator based on effective LOX inhibitors, further indicating potentially health-promoting effects for HT (Habza-Kowalska et al., 2019). Some studies have also shown that luteolin’s mechanisms showed potent anti-inflammatory activity, including the activation of NF-κB, which leads to the expression of IL-6 and COX-2 (Xagorari et al., 2001; Ching-Chow et al., 2004). Additionally, kaempferol, a polyphenol, is a bioactive substance with antioxidative, antimutagenic, antibacterial, and antiviral activities.

The KEGG enrichment analysis revealed that the chief pathways were concentrated in the AGE-RAGE signaling pathway in the pathogenesis of diabetes and its complications, the TNF signaling pathway, the PI3K–Akt signaling pathway, pathways in cancer, the mitogen-activated protein kinase (MAPK) signaling pathway, and some related to thyroid cancer. The present study identified JUN, AKI, MAPK1, and TP53 hub genes using the PPI network analysis. These results indicated that *PV* affected HT through the following pathways. Furthermore, a GO functional analysis also demonstrated many biological processes, including the inflammatory response, the cell-to-cell reaction, and the metabolic process in the present study. The TNF signaling pathway plays an essential functional role in regulating the inflammatory response (Noack and Miossec, 2017). Luteolin acts as an anti-inflammatory agent by regulating the TNF signaling pathway (Zhang et al., 2018; Zhang et al., 2019). A previous study demonstrated that the MAPK signaling pathway was revealed to be correlated with the progression of HT (Luo et al., 2018). Several studies have exhibited significant anti-inflammatory activity effects by inhibiting the MAPK signaling pathways (Chen et al., 2016; Wei et al., 2016). Luteolin is a common flavonoid that exhibits intense anti-inflammatory activity through the MAPK signaling pathway (Aziz et al., 2018). The key target gene of *MAPK1* was involved in the MAPK signaling pathway.

Meanwhile, the MAPK signaling pathway plays an important role in developing thyroid carcinoma, including cell proliferation and cell survival. The PI3K/AKT signaling pathway plays a key role in regulating the activation of inflammatory response cells and releasing inflammatory transmitters to the chronic inflammatory response in HT (Li et al., 2018). By regulating the PI3K–AKT signaling pathway, luteolin might be the critical pathway against inflammation, thus realizing the treatment of HT (Huang et al., 2020). Kaempferol-activated PI3K/AKT signaling exerts anti-inflammatory effects (Imran et al., 2019; Harikrishnan et al., 2020; Jantan et al., 2021).

The present study included some limitations. First, some further verified experiments need to be conducted. Second, their specific molecular docking methods and locations need to be verified by further experiments. A comprehensive understanding of the key gene target of *PV* and HT is key towards therapeutic discovery and development.

## Conclusion

In summary, the network pharmacology and molecular docking show that luteolin and kaempferol were the main active components of *PV*, which indicated that they might play an essential role in treating HT. *PV* may act against HT mainly through the TNF signaling pathway, the MAPK signaling pathway, and the PI3K–Akt signaling pathway.

## Data Availability

The raw data supporting the conclusions of this article will be made available by the authors, without undue reservation.
